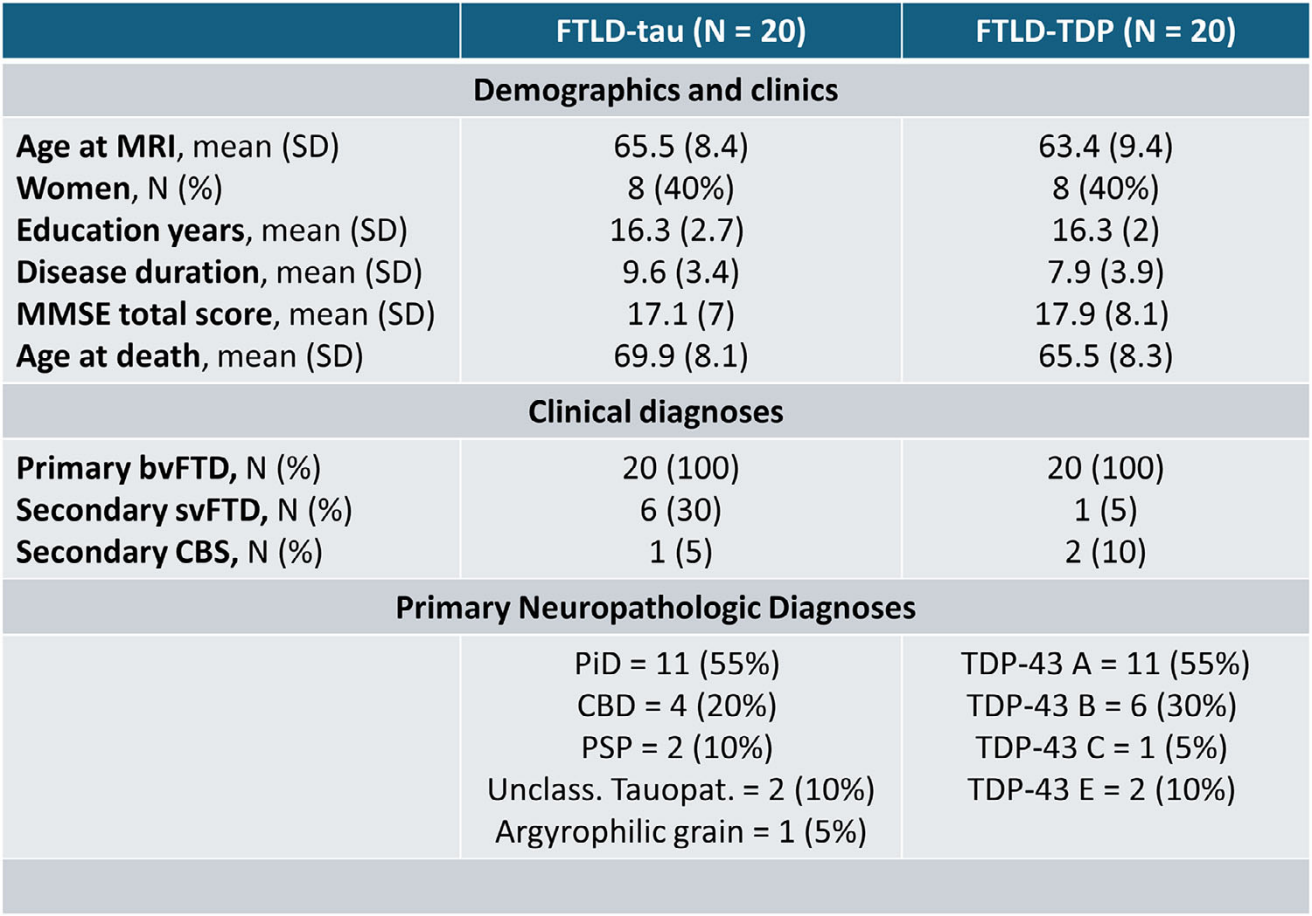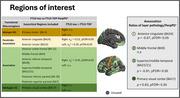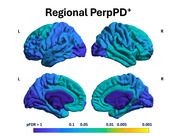# Investigating laminar distribution of tau or TDP‐43 pathology in frontotemporal lobar degeneration using cortical diffusion MRI

**DOI:** 10.1002/alz70856_107152

**Published:** 2026-01-08

**Authors:** Mario Torso, Gerard R Ridgway, Hamsi Radhakrishnan, Daniel T Ohm, Eddie B. Lee, Pegah Khosropanah, Ian Hardingham, Steven A Chance, David J. Irwin

**Affiliations:** ^1^ Oxford Brain Diagnostics, Oxford, United Kingdom; ^2^ Penn FTD Center, University of Pennsylvania, Philadelphia, PA, USA; ^3^ Penn Frontotemporal Degeneration Center, Department of Neurology, Perelman School of Medicine, University of Pennsylvania, Philadelphia, PA, USA; ^4^ Department of Pathology & Laboratory Medicine, Perelman School of Medicine, University of Pennsylvania, Philadelphia, PA, USA; ^5^ Penn Frontotemporal Degeneration Center, Department of Neurology, Perelman School of Medicine, University of Pennsylvania, Philadelphia, PA, USA

## Abstract

**Background:**

Frontotemporal dementia (FTD) can arise from frontotemporal lobar degeneration (FTLD) driven by distinct proteinopathies, such as tau (FTLD‐tau) or TDP‐43 (FTLD‐TDP), which can lead to remarkably similar clinical syndromes. Previous research (PMID:34997851) identified a predominance of tau pathology in the lower cortical layers and TDP‐43 pathology in the upper cortical layers. The aim of the present study was to investigate the effect of laminar distribution of pathology on cortical architecture using cortical diffusivity metrics.

**Method:**

Forty cases with a primary bvFTD clinical phenotype and autopsy confirmation from Penn Frontotemporal Degeneration Center were included in the study. The patients were grouped based on the primary neuropathological diagnosis: 20 FTLD‐tau and 20 FTLD‐TDP (Table‐1).

Structural and diffusion MRI (dMRI) were used to calculate whole‐brain and regional cortical diffusivity measures. For each cortical region and for four macroregions (idiotypic M1, paralimbic association, association and idiotypic V1) previously explored (PMID:34997851) (Figure 1), a minicolumn‐inspired cortical diffusivity measure that combines the components perpendicular to the radial minicolumns (PerpPD^+^) was calculated (PMID:36281682). Previous findings have shown that this measure is sensitive to tau neuropathology (PMID:37794477). For a subgroup of 17 cases, ratios of layer pathology (RLP) in four cortical regions (Figure 1) were generated.

Differences in diffusion metrics at regional and macroregional level were tested with a linear model adjusting for interval between MRI scan date and autopsy date, acquisition protocol, disease duration, age and sex, with false discovery rate correction (pFDR<0.05). Partial Spearman's rank correlation was conducted, including cases from both groups, to investigate the relationship between regional PerpPD^+^ and RLP values.

**Result:**

Regional analysis showed a significant pattern of higher PerpPD^+^ values in FTLD‐tau group, involving mainly fronto‐temporal regions (Figure 2). Macroregion comparisons revealed higher PerpPD^+^ in bilateral paralimbic and right association regions (Figure 1). Correlation analysis identified significant associations between ratio (RLP) values and PerpPD^+^ values in anterior cingulate, superior/middle temporal and primary visual cortex (Figure 1).

**Conclusion:**

Regional differences suggest that PerpPD^+^ can distinguish cortical microstructural changes in FTLD due to tau vs. TDP‐43. The correlation between PerpPD^+^ values and laminar pathology ratios reinforces that PerpPD^+^ is an MRI marker sensitive to tau pathology distribution.